# Malnutrition-Sarcopenia Syndrome: Is This the Future of Nutrition Screening and Assessment for Older Adults?

**DOI:** 10.1155/2012/651570

**Published:** 2012-09-13

**Authors:** Maurits F. J. Vandewoude, Carolyn J. Alish, Abby C. Sauer, Refaat A. Hegazi

**Affiliations:** ^1^Department of Geriatrics, ZNA St. Elisabeth Leopoldstraat 26, University of Antwerp, 2000 Antwerp, Belgium; ^2^Scientific and Medical Affairs, Abbott Nutrition, Abbott Laboratories, 3300 Stelzer Road, Columbus, OH 43219, USA

## Abstract

Malnutrition is common across varying patient populations, particularly older adults, and sarcopenia prevalence increases with advancing age. Both malnutrition and sarcopenia are associated with substantial adverse outcomes affecting both the patient and the healthcare system, including increased morbidity, mortality, rehospitalization rates, and healthcare costs. Healthcare practitioners may assess patients for either malnutrition or sarcopenia; however, many patients clinically present with both conditions, resulting in the syndrome, Malnutrition-Sarcopenia Syndrome, which is the clinical presentation of both malnutrition and accelerated age-associated loss of lean body mass, strength, and/or functionality. Clinicians are urged to screen, assess, and treat these conditions currently so as to adequately address the full spectrum of patients' nutritional issues. By examining aspects of both conditions, clinicians can more fully assess their patients' clinical and nutritional status and can tailor targeted therapies to meet their needs and improve outcomes. This proposed syndrome embodies the inherent association of malnutrition and sarcopenia, highlighting their combined impact on clinical outcomes. The objective of this review paper is to characterize Malnutrition-Sarcopenia Syndrome to advance clinical practice, by providing clinicians with the necessary background information to integrate nutritional assessment along with loss of muscle mass and functionality in their everyday clinical practice.

## 1. Introduction

Historically, malnutrition has been defined as a condition of an imbalance of energy, protein, and other nutrients that cause measurable negative effects on body composition, physical function, and clinical outcomes [1]. Typical measures that clinicians use to screen and assess for malnutrition or the risk for malnutrition include dietary or nutrient intake, changes in body weight, and laboratory values [2]. A new definition of malnutrition has recently been proposed by an International Guideline Consensus Committee, integrating the acuity of the associated disease and inflammation [3]. The committee specified three subtypes of malnutrition using an etiology-based terminology to assist clinicians to make a nutrition diagnosis in clinical practice settings: (1) starvation-related without inflammation, (2) chronic disease or conditions that impose sustained mild-to-moderate inflammation (e.g., sarcopenic obesity, organ failure, and pancreatic cancer), and (3) acute disease or injury states, when inflammatory response is marked [3].

**Figure 1:**
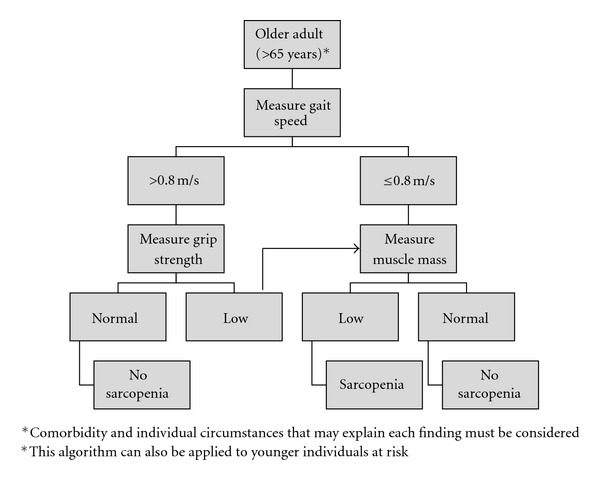
EUGMS working group-suggested algorithm for sarcopenia case finding in older individuals (used with permission) [9].

One critical clinical aspect often not assessed in nutrition screening or assessment is lean body mass or muscle mass loss. Lean body mass (LBM) is defined as that portion of the body mass that is everything but fat and includes water, mineral, muscle, and other protein-rich structures (e.g., enzymes, viscera, red cells, and connective tissues) [4]. Skeletal muscle mass constitutes the majority of LBM and provides strength, mobility, and balance [5]. Muscle mass also plays a critical role in whole-body protein metabolism and impacts quality of life in patients with chronic diseases [6]. The balance between muscle protein anabolism and catabolism is vitally important to maintaining skeletal muscle mass, particularly in older adults who lose muscle mass as a consequence of aging and/or illness [6, 7]. It was not until 1989, when Irwin Rosenberg introduced the term sarcopenia [8]. The European Working Group on Sarcopenia in Older People (EWGSOP) defines sarcopenia as an age-related loss of muscle mass, combined with loss of strength, functionality, or both (Figure 1) [9]. The working group also proposed a diagnostic algorithm for sarcopenia that is based on the presence of low muscle mass plus either low muscle strength (e.g., low handgrip strength) or low physical performance (e.g., 4 meter walking speed).

Sarcopenia is further classified into either primary or secondary categories. Primary sarcopenia, when no specific etiologic cause can be identified, is progressive and associated with the impact of aging: a reduction in motor neurons, alterations in skeletal muscle tissue including mitochondrial dysfunction, changes in the hormonal milieu (e.g., insulin resistance and a reduction in insulin-like growth factor-1 and an increase in proinflammatory cytokines, such as tumor necrosis factor *α* and interleukin 6. Next to the intrinsic, age-related processes, a multitude of extrinsic and behavioral factors can aggravate the development and/or progression of sarcopenia, leading to secondary sarcopenia, such as disuse and lack of physical activity, malnutrition, chronic inflammation, and comorbidity. As such, sarcopenia can be thought of as both a process and an outcome. Sarcopenia as a condition is a major cause of frailty and disability in older adults; as an active process, it is present in every person reaching adult life [9].

## 2. Malnutrition and Sarcopenia Are Prevalent among Older Adults

There are no published data demonstrating the co-occurrence of malnutrition and sarcopenia in older adults. However, research has shown that reductions in handgrip strength are common in individuals who have sarcopenia as well as in individuals who are malnourished [9, 10]. Many older adults are malnourished or at high risk for malnutrition due to many factors. Decreased appetite and food intake, poor dentition, an increased frequency and severity of acute and chronic medical conditions, multiple medications, social and economic challenges, and cognitive decline all play a role in the etiology of malnutrition among older adults. Advanced age is an independent risk factor for malnutrition and is associated with a lower body weight, body mass index (BMI), and serum albumin [11–13]. 

The prevalence of malnutrition is greater among older adults in health care settings than in the community (Table 1). In hospital settings, malnutrition among older patients is approximately 56% [11]. For older adults living in the community, the prevalence of malnutrition ranges from 1 to 10%, while 41–48% are at risk for malnutrition [14]. Consistently, in a large international study, malnutrition was present in 2% of those living in the community, 9% of outpatients and home care combined, 23% in the hospital, 21% in institutions, and 15% in those with cognitive impairment [15].

Age-associated loss of muscle mass is characterized by a 3–8% decline per decade after the age of 30 years with further decline in adults 60 years of age and older [7, 16]. With aging, the loss of muscle mass is accompanied by an increase in body fat [17]. On average, adults can experience annual losses of 0.23 kg of muscle and gains of 0.45 kg of fat between 30 and 60 years of age [18]. Acute illness and injury can accelerate age-related changes in body composition. Studies demonstrate that, following injury, patients can lose 5-6% of their total body weight—most of which is muscle mass —and gain between 4 and 11% in fat mass within 12 months, with the majority of LBM loss occurring within the first two to four months after the injury [19].

Using the EWGSOP diagnostic criteria, Landi et al. evaluated the baseline data of adults who were 80 years of age or older (*n* = 260) from the ilSIRENTE study. The results of the study indicated that sarcopenia is prevalent among community-dwelling older adults with no differences based on gender (25%) [20]. Using the same diagnostic algorithm, Landi et al. demonstrated that the prevalence of sarcopenia is slightly higher (32.8%) among older adults in long-term care settings and was higher among male residents (68%) than among female residents (21%) [21].

Reductions in muscle mass among older adults are common and can contribute to functional impairment, disability, increased risk for falls [20], lowered quality of life, and increased risk for mortality. For instance, using cross-sectional body composition data from the Third National Health and Nutrition Examination Survey, Janssen and colleagues determined the prevalence of class I sarcopenia (skeletal muscle mass index within one to two standard deviations below sex-specific values for young adults) and class II sarcopenia (skeletal muscle mass index two standard deviations of young adult values) in adults age 50 years of older. Among adults 50 years of age and older, the prevalence of class I sarcopenia was estimated between 37 and 47% in men and 50–61% in women, and a prevalence of class II sarcopenia between 5–7% in men and 7–11% in women. The prevalence of sarcopenia increased from the third to sixth decades and remained constant among subjects 70 years of age and older, while the prevalence of class I (59% versus 45%) and class II (10% versus 7%) was greater in women ≥50 years of age than men (*P* < 0.001). Moreover, functional impairment was 2-3-fold higher in older subjects with class II sarcopenia than those with normal muscle mass, even after adjusting for age, race, BMI, health behaviors, and comorbidities [22].

## 3. Malnutrition Sarcopenia Syndrome and Clinical Outcomes

In many patient populations, malnutrition and sarcopenia are present in parallel and manifest clinically through a combination of decreased nutrient intake, decreased body weight, along with a decrease in muscle mass, strength, and/or physical function. This leads us to coin the proposed clinical syndrome of Malnutrition-Sarcopenia Syndrome (MSS). MSS is the clinical presentation of both malnutrition and accelerated age-associated loss of lean body mass, strength, and/or physical performance. Malnutrition and sarcopenia are each independently associated with negative health consequences that impact older adults across health care settings. Patients with malnutrition and/or sarcopenia are at risk of increased morbidity and mortality, decreased quality of life and functioning and increased rehospitalization, length of hospital stay, and healthcare costs. Importantly, malnutrition and sarcopenia are associated with increased mortality [23–27]. Cederholm et al. found significant differences in mortality rates between malnourished patients and well-nourished patients after hospitalization (44% versus 18%, respectively) [23]. In community-dwelling older adults, unintentional weight loss and low BMI are associated with elevated 3-year mortality rates, and older adults reporting unintentional weight loss were 1.67 times more likely to experience mortality than those who reported no weight loss [28]. Newman and colleagues demonstrated that 5% weight loss over a three-year period is a significant and independent predictor of mortality in community-dwelling aging adults [17]. Similarly, Cereda et al. determined that low BMI predicted mortality in older institutionalized adults [29]. Interestingly, a prospective observational cohort of older adults demonstrated that higher lean mass and lean mass index predicted lower mortality with an 85% reduction in risk, suggesting that changes in lean mass and lean mass index, rather than BMI, are better predictors of mortality in older adults and highlighting the role of lean muscle mass loss in defining malnutrition [30]. Sarcopenia has also been linked with increased mortality in various patient populations. Recently, Landi et al. demonstrated that sarcopenia is highly prevalent in older nursing home residents and is associated with a significantly increased risk of all-cause death [31]. Additionally, Bunout et al. evaluated the relationship between the loss of fat-free mass and mortality among aging adults (*n* = 1413, 74.3 ± 5.6 years of age) and showed that low fat-free mass was significantly associated with mortality among individuals over 74 years of age [32]. Furthermore, results from a recent meta-analysis show that objective measures of physical capability, such as handgrip strength, walking speed, and chair rise, are predictors of all-cause mortality in older community-dwelling populations [33]. The loss of function associated with sarcopenia and malnutrition is a risk factor for negative outcomes.

Malnutrition and sarcopenia are associated with increased morbidity, in particular increased infection and complications rates, including falls [34] and disability [35]. In a study of hospitalized patients, malnutrition was shown to be a significant independent risk factor for nosocomial infections, with infection rates of 4.4% in the well-nourished group, 7.6% in the moderate malnutrition group as compared to 14.6% in the severely malnourished group [36]. In addition, serum albumin levels, age, weight, immunodeficiency, and nutrition risk index score were associated with increased risk of nosocomial infections [36]. Edington et al. determined that malnourished patients experience a two-fold increase in rates of infection as compared to well-nourished patients, indicating that malnutrition is associated with hospital-acquired infections [37]. Similarly, a study of hospitalized older adults demonstrated that patients identified with sarcopenia (detected by dual energy X-ray absorptiometry (DXA)) upon admission were at a greater risk of contracting a nosocomial infection during the first 3 weeks of hospitalization (relative risk of 2.1) [38].

Malnutrition and loss of muscle mass compromise the quality of life and functional capacity of aging adults. Malnutrition is associated with declines in functional capacity in hospitalized patients [39]. Moreover, reduced quality of life has been reported among malnourished patients with a total Mini-Nutritional Assessment (MNA) score <24 [40]. Malnutrition significantly impacts the clinical outcomes of community-dwelling older adults. Specifically, older patients who are malnourished are more likely to be discharged to a residential home and less likely to return home [41, 42] with an increased length of convalescence, greater disability and dependence on walking devices, and loss of muscle strength after hospitalization [43–45]. Other consequences of sarcopenia are persistent sense of fatigue, muscle weakness, increased predisposition to metabolic disorders, and increased risk of falls and fractures [46]. Studies suggest that loss of muscle mass is a predictor of functional decline in independent older adults and those with disability [22, 47] and that age-related loss of muscle mass is directly correlated with loss in strength [48]. Interestingly, Reid et al. showed that lower extremity muscle mass is a strong independent predictor of the level of functional impairment [49]. Loss of strength with aging tends to track with loss of muscle mass in physiological studies although the decline in muscle strength is steeper than the decline in muscle mass [50]. Also, interventions that increase muscle mass do not necessarily increase strength, and changes in strength that occur with resistance training precede measurable changes in muscle mass. Correlations between change in muscle mass and change in strength in older adults are therefore not consistent. Recently, the term ‘‘sarcopenia with limited mobility” was proposed as a syndrome that occurs when sarcopenia leads to loss of function and individuals become candidates for therapeutic interventions [51].

In addition to increased risk of infections and functional impairment, malnutrition and loss of muscle mass are also associated with increased hospital length of stay (LOS) [11, 37, 52–55]. Moreover, studies have shown that weight loss and malnutrition are predictors of increased rehospitalization rates in adults [56–59]. In one study, LOS is significantly shorter among well-nourished patients (5.7 days) as compared to malnourished patients (8.9 days) [60]. Leandro-Merhi et al. reported that well-nourished patients are three times more likely to be discharged sooner than patients with varying degrees of malnutrition [55]. Additionally, severely malnourished patients with a BMI < 20 kg/m^2^ or weight loss of greater than 10% stayed in the hospital even longer at 18.3 and 17.5 days, respectively [37]. Hospitalization is associated with significant reductions in muscle mass and strength and functional decline in older adults [44, 61–64]. Interestingly, in a large sample of patients, Pichard et al. determined that fat-free mass (FFM) and fat-free mass index are significantly lower among elderly hospitalized patients than their nonhospitalized counterparts. Additionally, 37% of patients hospitalized for just 1-2 days had low FFM, which increased to 55.6% after 12 days of hospitalization [65]. Another study concluded that short-term hospitalization was associated with significant declines in functional capacity and muscle strength, regardless of age or baseline functional status [45]. The relationship between hospitalization—related loss of muscle mass and strength and declining functional capacity and the risk for future hospital admission needs further exploration.

Collectively, malnutrition, loss of muscle mass, strength, and functional capacity are accelerated in hospitalized older adults [55, 61, 62, 66]. Increased LOS worsens malnutrition and sarcopenia, creating a vicious cycle of disease severity, increased frequency and severity of complications, increasing LOS, and rehospitalization rates [62].

Due to their associated comorbid conditions, malnutrition and sarcopenia impose a major economic burden on healthcare systems, contributing to escalating healthcare costs. A study of hospitalized patients showed significantly increased costs in malnourished patients. The mean daily expense was $228.00 per malnourished patients versus $138.00 per well-nourished patients, a cost increase of 60.5%, after including costs for medications and tests, the cost to treat malnourished patients rose by 308.9% [53]. According to a British Association of Parenteral and Enteral Nutrition report, malnutrition in the UK costs in excess of *£*13 billion per year: *£*8 billion is for healthcare, including hospital inpatients and outpatients, and primary care (prescriptions and general medical services), and *£*5 billion is for nursing, residential, and home care services [67]. Additionally, sarcopenia significantly increases health care costs. A 2000 US study estimated that sarcopenia resulted in $18.5 billion dollars in direct health care expenditures, which reflected 1.5% of total healthcare expenditures. Moreover, a 10% reduction in sarcopenia prevalence would result in $1.1 billion US dollars in health care savings [68].

## 4. Screening and Assessing for Malnutrition Sarcopenia Syndrome

Clinicians should integrate nutrition assessment with sarcopenia screening for optimal evaluation of these two inter-related nutritional issues to help improve patients' clinical outcomes. A variety of malnutrition screening tools are available such as the Malnutrition Screening Tool (MST) [69], Malnutrition Universal Screening Tool (MUST) [70], the short form of the Mini-Nutritional Assessment (SF-MNA) [71] and Nutrition Risk Screening-2002 (NRS-2002) [72]. A standard for nutritional assessment, the Subjective Global Assessment (SGA), is a valid and reliable method to assess nutritional status in a variety of patient populations [73]. The MNA is another reliable assessment tool validated for use with older adults in multiple settings [71, 74]. Elements of history and physical examination are commonly shared among these tools and include unintentional weight loss (e.g., 3 kg within the last 3 months), decreased food intake, gastrointestinal symptoms, and functional impairment. For sarcopenia screening, a simple clinician tool has been suggested by the European Geriatric Medical Society (EUGMS) Consensus Committee of defining sarcopenia (Figure 1), in which older adults are screened and assessed for sarcopenia using both gait speed and handgrip strength measurements [9, 75–77]. If a patient is identified to have slow gait speed or low hand grip strength, muscle mass should then be measured and evaluated. Based on the evidence presented, the combination of screening and assessing for malnutrition and sarcopenia is recommended to screen for the presence of MSS in at-risk patient populations, particularly older adults in clinical settings and in the community. The proposed clinical signs and symptoms to identify MSS are highlighted in Table 2. Specifically, to facilitate screening and assessment of MSS, we propose that patients would be at high risk for MSS if at least four of these criteria are present:

1. recent history of reduced appetite that resulted in poor food intake,
2. unintentional weight loss of 3 kg or more over the last 3 months,
3. low muscle mass (as measured by DXA, CT, MRI, or BIA),
4. decreased gait speed (less than 0.8 meter/second), and
5. reduced hand grip strength for age and gender.

Future research is warranted to determine the reliability and validity of this assessment tool across patient populations and settings.

## 5. Conclusion

Malnutrition and sarcopenia are both commonly occurring conditions across patient populations, especially older adults. Both conditions result in numerous and substantial negative outcomes to both the patient and the health care system, including increased morbidity and mortality, decreased patient quality of life and functionality, and increased health care costs and rehospitalization rates. Historically, patients have been screened or assessed by healthcare practitioners for either malnutrition or sarcopenia, but rarely for both conditions concurrently. However, many patients present clinically with both conditions in parallel and this combination, or the malnutrition sarcopenia syndrome should be the focus of future nutrition screening and assessment in at-risk patient populations. Examining the entirety of the patient's nutritional and functional status through screening and assessment for both malnutrition and sarcopenia will enable healthcare practitioners to better determine the presence of MSS in their patients and target interventions to fit the patients' needs. Moreover, as the world is aging and older adults will utilize health care services at an increased rate, this could ultimately result in better patient care and outcomes in this unique and expanding patient population. Clinicians and researchers are called upon to work together to develop a practical, reliable, and valid tool for MSS that is appropriate for implementation into a variety of clinical practice settings, with the aim of identifying patients with MSS and providing the appropriately targeted interventions.

## Figures and Tables

**Table 1:** Prevalence of malnutrition and sarcopenia in older adults across clinical settings.

Patient Population/Setting	Malnutrition	Sarcopenia
Hospital/Acute Care	56% [11]	
	23% [15]	
Long Term Care	21% [15]	32.8% [21]
	2–9% [15]	25% [20]
Community	1–10% [15]	37–61% (class I sarcopenia) [22]
	(41–48% at risk) [14]	5–11% (class II sarcopenia) [22]

**Table 2:** The clinical signs and symptoms of Malnutrition-Sarcopenia Syndrome.

Malnutrition-Sarcopenia Syndrome	
Malnutrition	Sarcopenia
↓ Food intake	↓ Muscle mass
↓ Appetite	↓ Muscle strength and/or functionality
↓ Body weight	
